# 1.0 s Ultrafast MRI in non-sedated infants after reduction with spica casting for developmental dysplasia of the hip: a feasibility study

**DOI:** 10.1007/s11832-016-0734-8

**Published:** 2016-04-13

**Authors:** Atsushi Fukuda, Kenichi Fukiage, Tohru Futami, Tosiaki Miyati

**Affiliations:** Department of Radiology, Shiga Medical Center for Children, 5-7-30 Moriyama, Moriyama, Shiga 524-0022 Japan; Department of Pediatric Orthopaedics, Shiga Medical Center for Children, 5-7-30 Moriyama, Moriyama, Shiga 524-0022 Japan; Division of Health Sciences, Graduate School of Medical Sciences, Kanazawa University, 5-11-80 Kodatsuno, Kanazawa, Ishikawa 920-0942 Japan

**Keywords:** Ultrafast magnetic resonance imaging, Developmental dysplasia of the hip, Spica casting, Reduction, Femoral head location

## Abstract

**Purpose:**

The aim of this study was to first develop and use 1.0 s ultrafast magnetic resonance imaging (MRI) to confirm the location of the femoral head in non-sedated infants with developmental dysplasia of the hip (DDH) after reduction with spica cast application in clinical settings.

**Methods:**

The ultrafast acquisition was achieved by employing a balanced steady-state free precession sequence and immobilizing the patient with dedicated sandbags. On completion of the ultrafast MRI study, all infants were sedated for conventional MRI scanning. Two orthopaedic surgeons retrospectively evaluated the image quality, result of the reduction and total MRI study time (including patient immobilization, coil setup, and scanning) in 14 DDHs of 13 infants (one with bilateral DDHs).

**Results:**

Both reviewers stated that there were no motion artefacts for non-sedated infants during the ultrafast MRI and that the quality of both the ultrafast and conventional MRI images were acceptable to assess the femoral head location. Assessment of the reduction procedure resulted in two hips being categorized as ‘incomplete reduction’ requiring a re-reduction procedure. The total study time of ultrafast and conventional MRI was 6 ± 1 min and 14 ± 3 min, respectively (*P* < 0.001). No complications due to sedation, such as hypoxia, were reported. The average sedation waiting time was 1 h 25 min ± 34 min.

**Conclusion:**

The ultrafast MRI procedure reported here can be readily employed to confirm the location of the femoral head in infants with DDHs, without the use of any sedation.

## Introduction

Developmental dysplasia of the hip (DDH) treatment consists of initial concentric reduction followed by stabilization. Several reduction techniques are commonly used, such as the Pavlik harness [1], acute manual closed reduction [2], gradual reduction by traction [3] and open reduction [4]. In all of these procedures, confirmation of concentric reduction and monitoring of the concentricity during the stabilization phase are imperative. Radiography [5], ultrasonography [6], computed tomography (CT) [7] and magnetic resonance imaging (MRI) [8] are the standard imaging modalities used to assess the location of the femoral head.

Radiography has been the preferred diagnostic method to diagnose DDH due to the benefits of less radiation exposure, greater convenience and low cost. However, radiography has the disadvantage that it does not enable easy confirmation of the relation between the cartilaginous femoral head and acetabulum in spica cast application. Using ultrasonography it is possible to image the cartilaginous femoral head, and this modality is reported to be reliable when compared with radiography [5]. However, care is necessary because false-positive findings can occur if the imaging plane is suboptimal. In cases evoking doubt, CT and MRI are used to determine the exact position of these hips [8, 9].

MRI can also provide a high tissue contrast when used for infants with DDH, does not expose the infant to radiation and enables assessment of the femoral hip location in two orthogonal planes (transverse and coronal views) to prevent misdiagnosis [8, 10, 11]. However, because conventional MRI is particularly sensitive to motion artefacts, the infant may need to be sedated [12]. In addition, MRI is an expensive and time-consuming process. Consequently, a useful alternative is of interest.

To solve these problems, we have developed and employed a 1.0 s ultrafast MRI protocol to confirm the location of the femoral head in non-sedated infants with DDH after reduction with spica cast application in the clinical setting. The acquisition was achieved by employing a balanced steady-state free precession sequence (bSSFP) [13] and immobilizing the patient using dedicated sandbags. Thus, the aim of this study was to assess the validity of ultrafast MRI by comparing its efficacy with conventional MRI in the same patient population.

## Materials and methods

This was a retrospective study and was approved by the institutional review board of the Shiga Medical Center for Children. Written informed consent was obtained from the parents or guardians of the infants received sedation and participated in the MRI examination.

### Study population

We consecutively evaluated 13 infants (12 girls, 1 boy) retrospectively after spica cast application from April 2015 to October 2015. The average age during scanning was 6.9 (range 3–16) months. There were 11 infants with left DDH, one with right DDH and one with bilateral DDH.

### Gradual reduction

All infants were treated by ultrasound-guided gradual reduction using flexion and abduction continuous traction (FACT-R), which comprises five steps [3]. The first step consists of longitudinal indirect skin traction for a period of more than 2 weeks with the hip in 20° of flexion and the knee extended. The second step consists of indirect lateral skin traction in abduction and flexion to release the contracture of the adductor muscles and deliver the femoral head from behind the acetabulum. The traction is removed at least twice a day during the first two steps for skin care purposes. The third step is the reduction phase during which the weight of traction is reduced. The children remain in the hospital during the first three steps, but they are allowed home overnight or for weekends during the first and second steps depending on the treatment results. The fourth step is the spica casting to maintain the reduction, and the fifth step is the conversion of the spica cast to a Pavlik harness or abduction brace.

### Imaging

**Fig. 1:**
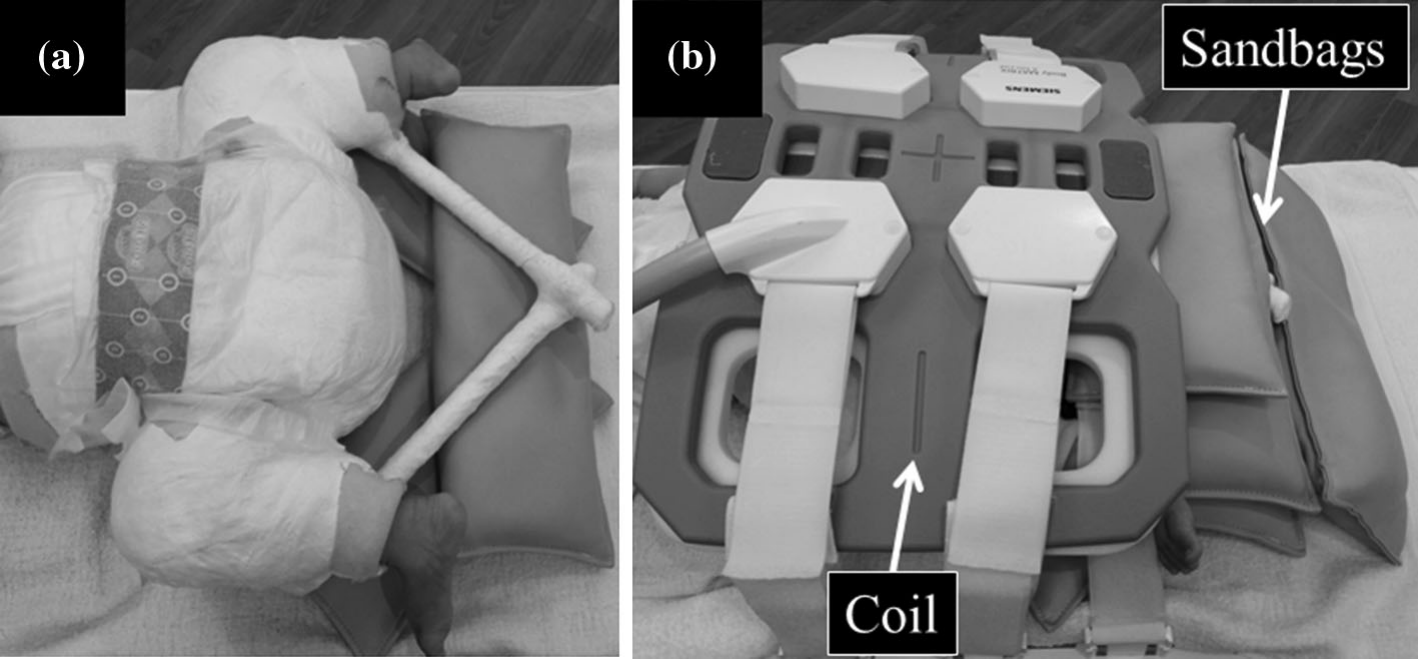
Photographs of the system used to immobilize patient for magnetic resonance imaging (MRI), before (**a**) and after immobilization (**b**). Infants were placed on the examination table and were immobilized using MRI dedicated sandbags and coil

The MRI examinations were conducted using a 1.5T MRI system (Avanto; Siemens, Erlangan, Germany). Immediately after reduction with spica casting (step four in FACT-R), we performed the ultrafast MRI examination to confirm the location of the femoral head using neither sedation nor general anaesthesia. MRI technologists carefully immobilized the infants with spica cast application on the scanning table using MRI dedicated sandbags and coil (Fig. 1). Using the bSSFP sequence, the ultrafast MRI images were acquired with a repetition time/echo time of 6.16/2.6 ms, a flip angle of 30°, slice thickness of 3 mm and a scanning time of 11 s per 11 slices. This sequence can acquire the image like a ‘snapshot’ (one image per 1 s) and tolerate patient motion.

On completion of the ultrafast MRI examination, all infants were administered triclofos syrup (20–80 mg/kg body weight) and/or chloral hydrate (30–50 mg/kg body weight) for the second conventional MRI scan. We then employed the multiple-echo data image combination (MEDIC) sequence with and without fat-saturation [14] and double-echo steady-state (DESS) pulse sequence. The scanning times were 1:54, 2:47 and 1:48, respectively.

### Qualitative analysis

All ultrafast MRI and conventional MRI studies were anonymized and independently evaluated by two paediatric orthopaedic surgeons. These investigators assessed the quality of the ultrafast MRI and conventional MRI images using a three-point scale based on the relationship between the femoral head and acetabulum. The ultrafast MRI results were scored first, and the conventional MRI results were scored at least 2 weeks later. A score of 1 was assigned when the image quality was considered to be non-diagnostic (poor) due to the lack of contrast or severe motion artefact. A score of 2 was assigned when the delineation of anatomical landmarks was unclear, but a diagnosis was still possible (adequate). A score of 3 was assigned when the delineation of landmarks was excellent, and its assessment was practical (excellent). The results of each reviewer were tabulated, and a consensus reading was performed. These surgeons then evaluated each hip as a ‘normal/reduced hip’ or ‘incomplete reduction (re-reduction procedure is required)’.

### Statistical analysis

The Shapiro–Wilk test for normality and Wilcoxon signed-rank test were used to calculate the statistical differences of the data obtained. Inter-observer agreement regarding the degree of image quality and inter- and intra-observer agreement of the result of the reduction procedure were determined using the kappa (*κ*) coefficient. Interpretation of the strength of *κ* was as follows: a score of 0.40–0.60 indicated moderate agreement; 0.60–0.80, substantial agreement; >0.80, almost perfect agreement. A *P* value of ≤0.05 was considered to indicate statistically significance. All statistical analyses were computed using R version 3.0.3 for Windows software package.

## Results

**Fig. 2:**
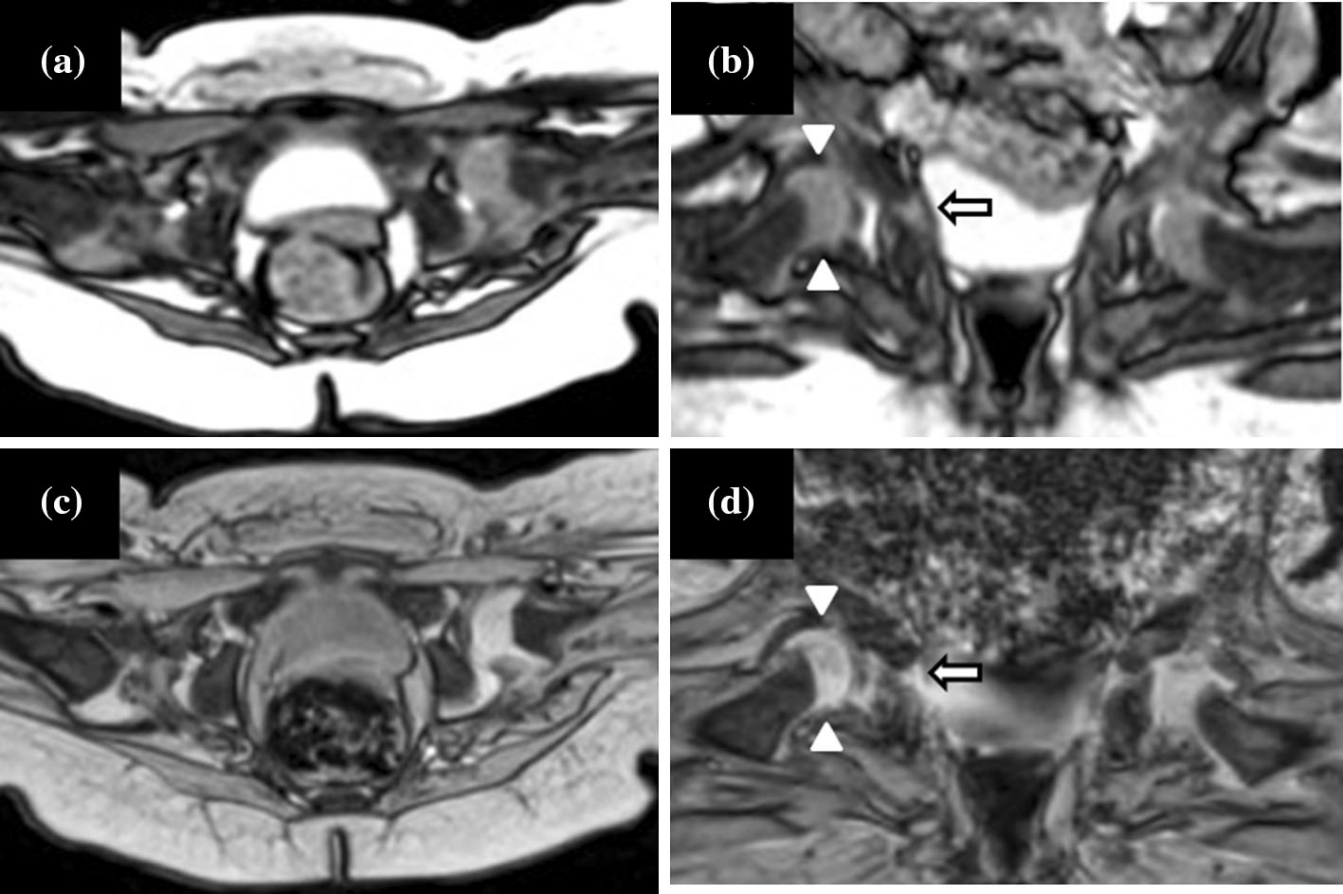
Transverse and coronal images by ultrafast (**a**, **b**) and conventional MRI (MEDIC) (**c**, **d**) of an incomplete reduction of the right hip and reduced left hip in an 8-month-old girl after the reduction procedure. No motion artefact was observed on ultrafast MRI. The results of the transverse and coronal images of the ultrafast MRI examination are similar to those of the conventional MRI examination. *White arrow* and *arrowheads* Right triradiate cartilage and incomplete reduction of the right hip, respectively. MEDIC Multiple-echo data image combination

**Fig. 3:**
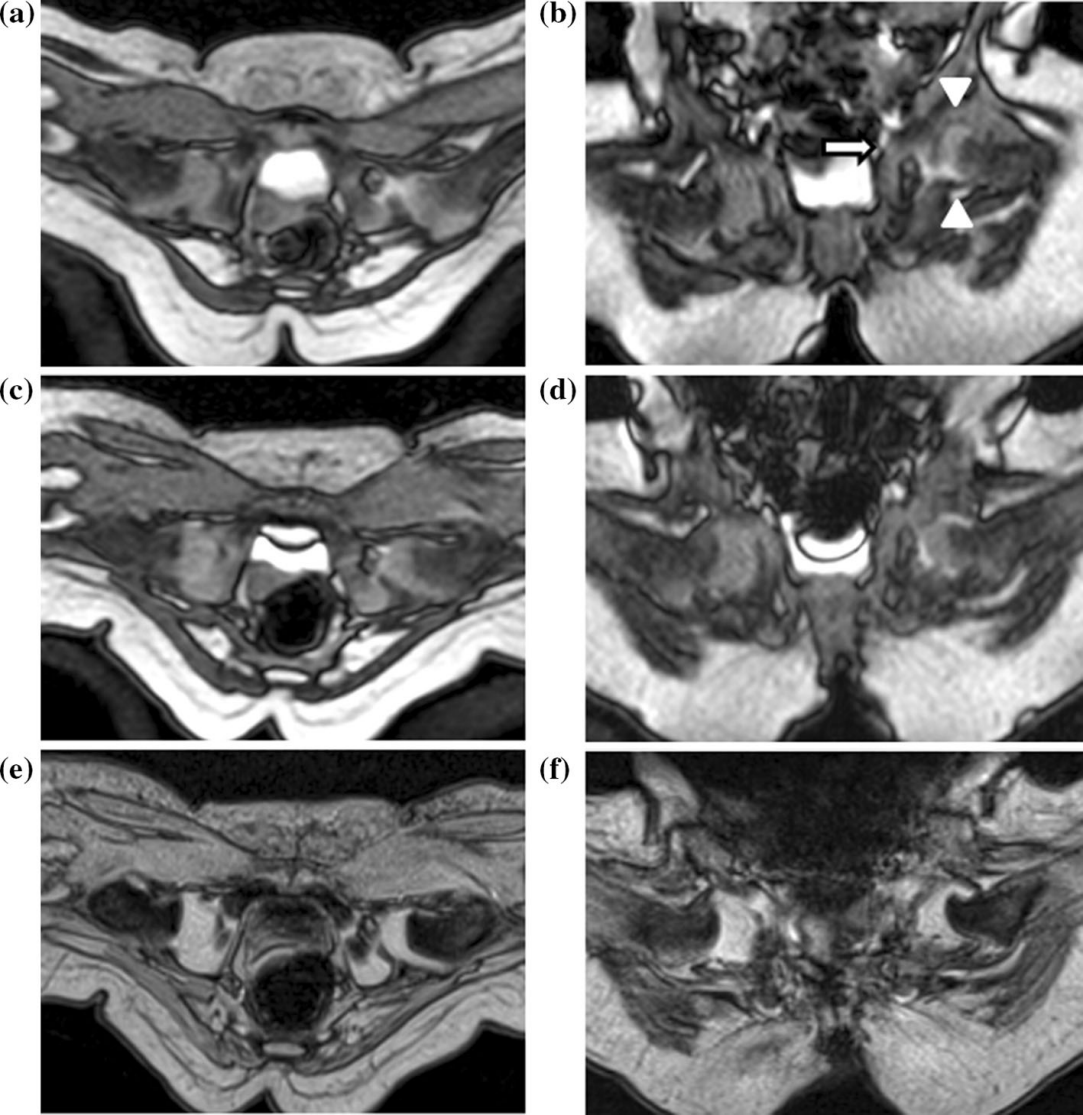
**a**, **b** Transverse and coronal images of the first ultrafast MRI study revealed an incomplete reduction of the left hip in a 6-month-old girl after gradual reduction with spica casting.* White arrow* and* arrowheads* Left triradiate cartilage and incomplete reduction of the left hip, respectively. The re-casting procedure was performed immediately after imaging. **c**–**f** Transverse and coronal images of the second ultrafast (**c**, **d**) and conventional MRI (MEDIC) (**e**, **f**) show successful reduction of the left developmental dysplasia of the hip. No motion artefact was observed on the ultrafast MRI

The novel ultrafast MRI procedure was employed to confirm the location of the femoral head in all 13 infants participating in the study (14 DDHs, 12 normal hips) after casting. In one infant with bilateral DDH, the reduction was performed in another hospital, and the infant then transferred to our clinic for confirmation of the location and stability of the femoral head. Transverse and coronal ultrafast MRI demonstrated an incomplete reduction of the right hip and left reduced hip in this infant (Fig. 2), which was confirmed by conventional MRI. Because gradual reduction with spica casting was performed for this infant at our hospital, this study includes two sets of MRI studies for this infant. Another infant was diagnosed with an incomplete reduction of left hip after casting by ultrafast MRI (Fig. 3a, b). Because the re-casting procedure was immediately performed for this patient, conventional MRI was not used. After re-casting, the second ultrafast MRI (Fig. 3c, d) and conventional MRI (Fig. 3e, f) revealed a reduced left hip in this infant. Therefore, in this study, ultrafast MRI and conventional MRI were used for 15 and 14 examinations in 13 infants (14 DDHs and 12 normal hips), respectively.

Our records show that the mean study times (from enter to exit of the MRI scanning room) of ultrafast MRI and the conventional second MRI were 6 ± 1 and 14 ± 3 min, respectively (*P* < 0.001). No complications due to sedation, such as hypoxia, were reported in this patient population (Table 1). The average sedation waiting time was 1 h 25 min ± 34 min. No sedation was required for three infants because they fell asleep during the ultrafast MRI examination.

**Table 1:** Magnetic resonance imaging study times, sedation waiting times and the image quality scores of infants with developmental dysplasia of the hip

MRI modality	Study time (min)	Sedation waiting time (min)	Image quality score
Ultrafast MRI	6 ± 1 (4–8)	NA	2.9 (2–3)
Conventional MRI	14 ± 3 (11–24)	85 ± 34 (48–166)	3.0 (3–3)
*P* value	*P* < 0.001	NA	NS (*P* = 0.125)

Values in this table are presented as the mean ± standard deviation with the range given in parenthesis

*MRI* Magnetic resonance imaging, *NA* not applicable, *NS* no significant difference

Both reviewers stated independently of each other that there were no motion artefacts for non-sedated infants during the ultrafast MRI examination and that the quality of both the ultrafast MRI and conventional MRI images was acceptable to assess the location of the femoral head in infants with spica cast application after the reduction procedure (Table 1). Inter-observer agreement (*κ*) was 0.782 (substantial agreement).

The results of the reduction procedure show that two hips were categorized as ‘incomplete reduction’, thereby requiring a re-reduction procedure (Table 2; Figs. 2, 3). Inter- and intra-observer agreement (*κ*) were both 1.0 (almost perfect agreement).

**Table 2:** The result of reduction procedure

Conventional MRI	Ultrafast MRI	
	Normal/Reduced hip	Incomplete reduction
Normal/reduced hip	28	0
Incomplete reduction	0	1/2^a^

^a^Ultrafast MRI: 2, Conventional MRI; 1, conventional MRI was not applied in one case

## Discussion

Closed reduction with spica cast application is conventionally performed under general anaesthesia. This procedure has become an important and widely accepted procedure because adductor muscle tenotomy or arthrography can be concurrently performed in the operating room [15]. Location of the femoral head can subsequently be confirmed by three-dimensional (3D) fluoroscopy [16]. Although hip location in spica cast can be confirmed using these techniques during the procedure, MRI may be applied without additional general anaesthesia or sedation to assess femoral head location [17, 18]. However, the major drawback of this procedure is the general anaesthesia used in the operating room.

Gradual reduction by traction with spica cast application has been reported as an alternative reduction procedure [3]. Because general anaesthesia or sedation is not required to perform this type of procedure, its use in the infant patient population has clear benefits. In this procedure, ultrasonography is conventionally used to confirm concentricity of the femoral head. Although ultrasonography is a convenient and repeatable technique, the acoustic window is limited due to the presence of the spica cast [5] and, consequently, false-positive findings can occur if the imaging plane is suboptimal. In these cases, if general anaesthesia or sedation is not required for the MRI study, MRI is a powerful technique for assessment of the location of the femoral head. Desai et al. reported that spica MRI could be performed without sedation by soothing or swaddling the infant in a blanket [18]. However, spica casting does not eliminate the motion artefact altogether. Many infants become agitated during the MRI examination even with soothing from parents or guardians. As a result, conventional MRI is not a useful diagnostic modality to image the femoral head and acetabulum without motion artefact. Therefore, the most effective strategy is to perform the imaging study when the infant is asleep or our new ultrafast acquisition.

Temporal resolution refers to the time that is needed to acquire one image, making it the most important factor in the imaging procedure when the aim is to reduce the motion artefact. The temporal resolution of CT is considered to be higher than that of MRI, being approximately 0.5 s for an abdominal CT [19, 20]. Although CT enables the image to be acquired without a motion artefact, it may not be practical for imaging infants due to its relatively higher radiation dose and lower tissue contrast [21]. With recent developments in MRI scanning, the bSSFP MRI sequence has been developed and released onto the commercial market [13]. This method is tolerant of patient motion due to the high temporal resolution of the sequence (48–64 ms) [22]. We have customized the bSSFP sequence to have a higher contrast between the cartilage and acetabulum with a temporal resolution of 1.0 s. This temporal resolution is comparable to that of abdominal CT and is 100-fold higher than that of the conventional MRI sequence (1.0 s vs. 1 min 54 s on MEDIC). In our study, the higher temporal resolution definitely contributed to the successful capture of the image of the non-sedated infant without motion artefact (Figs. 2, 3).

MRI is a conventionally time-consuming imaging modality, but one of its major drawbacks for application in the infant population is the length of the total study time. However, our proposed ultrafast MRI offers a short study time of ≤7 min, including patient immobilization, coil setup and scanning. In addition, our method was not only able to confirm concentric reduction after gradual reduction but could also monitor the concentricity during the stabilization phase of DDH.

We believe that knowledge of the ultrafast MRI sequence is useful to paediatric orthopaedic surgeons for the following reasons. First, ultrafast MRI can confirm the location of the femoral head without any motion artefact in non-sedated infants after the reduction with spica cast application, with an acquisition time of only 1.0 s per one axial or coronal image. In other words, no sedation or general anaesthesia is required for confirmation of the concentricity of the femoral head after spica cast application. Second, if the ultrafast MRI result shows pseudo-reduction, the re-casting procedure can be performed immediately after the imaging study (Fig. 3). Furthermore, ultrafast MRI can be performed after the re-casting procedure as needed because no sedation is required for the imaging. Third, the ultrafast MRI study would be beneficial in a busy radiology department because of the total study time of ≤7 min (including time of immobilization, coil setup and scanning). Lastly, the ultrafast bSSFP MRI sequence is commercially available through the recently released MRI system.

This study has two limitations. We are currently employing the ultrafast MRI to confirm location of the femoral head in clinical practice just after reduction with spica casting (fourth step in FACT-R). Although the contrast in ultrafast MRI is sufficiently efficient to confirm the location of the femoral head, it is definitely inferior to that of conventional images (Figs. 2, 3). Consequently, it was impossible to conduct a truly blinded analysis. Moreover, ultrafast MRI cannot detect the inverted labrum due to tissue contrast and spatial resolution. Consequently, it cannot replace conventional MRI in our hospital. Second, our study had retrospective design, and only 13 infants were analysed. Therefore, further research may be required to verify the usefulness of ultrafast MRI in confirming the location of the femoral head. Despite these shortcomings, we believe that ultrafast MRI is an effective imaging technique for non-sedated infants for confirmation of the location of the femoral head because there is no need for general anaesthesia or sedation.

In conclusion, we developed a 1.0 s ultrafast MRI to confirm the location of the femoral head in non-sedated infants with DDH after gradual reduction with spica cast application. The ultrafast MRI sequence provides excellent image quality without motion artefacts within a total study time of ≤7 min.
